# Dominance of an alien shrub *Rhus typhina* over a native shrub *Vitex negundo* var. *heterophylla* under variable water supply patterns

**DOI:** 10.1371/journal.pone.0176491

**Published:** 2017-04-26

**Authors:** Ning Du, Xiangfeng Tan, Qiang Li, Xiao Liu, Wenxin Zhang, Renqing Wang, Jian Liu, Weihua Guo

**Affiliations:** 1 Institute of Ecology and Biodiversity, School of Life Science, Shandong University, Jinan, Shandong Province, China; 2 Shandong Provincial Engineering and Technology Research Center for Vegetation Ecology, Shandong University, Jinan, Shandong Province, China; 3 Institute of Environmental Research, Shandong University, Jinan, Shandong Province, China; Fudan University, CHINA

## Abstract

Temporal heterogeneity of a resource supply can have a profound effect on the interactions between alien and native plant species and their potential invasiveness. Precipitation patterns may be variable and result in a higher heterogeneity of water supply with global climate change. In this study, an alien shrub species, *Rhus typhina*, introduced to China from North America and a native shrub species, *Vitex negundo* var. *heterophylla*, were grown in monoculture and mixed culture under different water supply regimes, with four levels of water supply frequencies but with a constant level of total supplied water. After 60 days of treatments, the alien species was found to be the superior competitor in the mixed culture and was unaffected by changes in the water supply pattern. The dominance of *R*. *typhina* was mainly owing to its greater biomass and effective modulation of leaf physiology. However, in the mixed culture, *V*. *negundo* var. *heterophylla* exhibited both leaf- and whole-plant-level acclimations, including higher leaf length to petiole length and root to shoot biomass ratios, and lower specific leaf weight and leaf length to leaf width ratio. Plant height of *V*. *negundo* var. *heterophylla* was comparable to that of *R*. *typhina* in the mixed culture, which is a strategy to escape shading. Although water treatments had little effect on most traits in both species, the possible influence of water regimes should not be neglected. Compared with high-frequency water supply treatments, more individuals of *V*. *negundo* var. *heterophylla* died in low-water-frequency treatments when in competition with *R*. *typhina*, which may lead to species turnover in the field. The authors recommended that caution should be exercised when introducing *R*. *typhina* to non-native areas in the context of global climate change.

## Introduction

Alien species have potentially significant effects on local ecosystems, including population dynamics, community composition, and ecosystem function [1, 2, 3]. The structure and function of local ecosystems can change entirely if the alien species exert high pressure on the native dominant species in a local area. Significant differences between the leaf traits of co-occurring alien and native species have been reported. For example, alien species have been demonstrated to have a higher leaf nitrogen (N) content, leaf phosphorus (P) content, photosynthetic capacity, specific leaf area, leaf area ratio, higher nitrogen allocation to photosynthesis versus cell walls, and lower leaf life span [4, 5, 6]. Most of the traits are associated with rapid carbon capture and fast relative growth rate. However, in low-resource environments, plant traits associated with high resource-use efficiency favor alien species colonization [2]. It is important to understand the mechanisms by which alien species outperform native species, because when the alien plants occupy the growth space of natives and produce reproductive offspring in very large numbers without direct intervention by humans, they will have the potential to disperse over a considerable area and classify as invasive species [7]. However, relatively few studies have focused on pair-wise competition of alien and native species, especially in the context of changing resource supply patterns (but see [8, 9]).

Temporal heterogeneity of water supply often occurs in the field. In the context of global climate change, the precipitation pattern may be variable and result in more water supply heterogeneity, which is a considerable challenge faced by terrestrial ecosystems [10, 11, 12]. Longer periods between rainfall events and a tendency for lower precipitation during summer are expected in some regions [13, 14], such as northern China. Strong precipitation variability was observed in different parts and seasons of Asia [15]. Resource heterogeneity and availability are both important for plant growth and assemblage responses [10, 13]. For the same total water input, a water supply with high frequency can be more homogeneous than one with low frequency. Compared to total water availability, a pulsing regime has much stronger effects on relative competitive abilities and, thus, may be more likely to influence field distribution patterns and plant community structures [11, 16]. Therefore, understanding how different species may be affected by temporal variation in water supply is imperative [13]. Previous studies have noted that temporal heterogeneity of water supply can alter the survival, growth, and productivity of plants, even when the same total amount of water is provided [12, 13, 16]. However, no consistent results have been obtained concerning biomass accumulation and allocation [12]. In addition, few studies have measured leaf functional trait acclimation with the interaction of competition and water heterogeneity supply treatment. In addition, competitive pressures can reduce or magnify the direct responses of a focal species to resource heterogeneity; therefore, the responses of focal species to the temporal heterogeneity of water supply should be studied in relation to competition from neighboring species.

Interactions with native species are crucial to determine the invasiveness of an alien species, and the impact of water supply heterogeneity on the competitive effects of alien species is among the key issues that need to be understood. A native shrub species, *Vitex negundo* var. *heterophylla*, and an exotic shrub species, *Rhus typhina*, were selected in this study. *V*. *negundo* var. *heterophylla* is a deciduous dominant shrub native to China, which belongs to Verbenaceae. It is drought-tolerant and a typical pioneer species, which is distributed in a broad range of habitats in northern China [17]. *R*. *typhina*, a deciduous member of Anacardiaceae, is a large shrub or a small tree native to eastern North America [8, 18, 19], which is highly invasive in Europe [20, 21]. However, there has been controversy about whether it should be identified as an invader species in China since its introduction in 1959 [3, 19]. *R*. *typhina* effectively retains water and soil, which confers some ecological benefits. This species has been utilized widely for afforestation in China owing to its vigorous growth under harsh conditions. It is also valued for its bright red foliage in autumn [18] and the air cleaning capacity [22]. However, it is a potential threat to the native species and local ecosystems since *R*. *typhina* has an aggressive growth strategy and is highly competitive as a clonal shrub [3]. It has formed highly dense monospecific populations through agamogenesis [18], and displayed strong invasive abilities over the other species. In our previous study, *R*. *typhina* was found to be more dominant than the native woody species *Quercus acutissima*, regardless of the soil N:P ratios [8]. The growth of two other native species (*Cotinus coggygria* and *Platycladus orientalis*) has also been suppressed by the presence of *R*. *typhina* [19]. In this research, both plant species are early-successional species, and now coexist in many areas on hills (personal observation) in northern China, which provides a good model to study species interactions as the plants overlap more and the competition is more intense in early successional plant communities [23].

In our study, seedlings of *V*. *negundo* var. *heterophylla* and *R*. *typhina* were grown in monocultures and mixed cultures with different water supply regimes. Leaf, stem, and whole plant growth traits were measured to determine: i) which species will be dominant in the competition and ii) whether the water supply pattern will affect the results of this competition. As the alien species will possibly have invasive abilities in fluctuating environmental conditions [24], we hypothesized that the competition ability of *R*. *typhina* would increase with low water supply frequency. The main objective of the present study was to enhance the understanding of plant invasiveness to temporal water supply heterogeneity, which will occur more often with global climate change.

## Materials and methods

### Plant material and growth conditions

The experiment was conducted in a greenhouse at the Fanggan Research Station of Shandong University, Shandong Province, China (36°26' N, 117°27' E). The maximum photosynthetic photon flux density in the greenhouse reached 1,600 μmol m^-2^ s^-1^ during the course of the experiment. Air temperature varied between 20 and 30°C and the relative humidity varied between 70 and 95% during the treatment.

Seeds of *R*. *typhina* and *V*. *negundo* var. *heterophylla* were collected from semi-natural hills around the station in October 2010, air-dried, and stored at 0–4°C throughout the winter. In May 2011, seeds were soaked for 24 h before being germinated on moist pledgets. Healthy and uniformly germinated seeds were selected and sown on June 1^st^. Seeds of both species were germinated at approximately the same time and used in different cultivations. In the monoculture treatment, one seed was sown in each pot, and in the mixed culture treatment, the seeds of each species were sown about 10 cm apart.

Two different sizes of pots were used in the experiment; the larger ones (32 cm tall, 29 cm upper diameter, and 25 cm lower diameter) were used for the mixed culture, and the smaller ones (21 cm tall, 20 cm upper diameter, and 15 cm lower diameter) were used for the monoculture. Sandy loam and humic soils were air-dried and mixed (2:1, v/v) as a growth substrate. The soil mix in the smaller pots (5 kg in dry weight) was half of that in the larger ones (10 kg in dry weight), and the soil had a pH of 5.9, and averaged 16.4 g kg^-1^ organic matter, 1.11 g kg^-1^ nitrogen, 1.12 g kg^-1^ phosphorus, and 23.1 g kg^-1^ potassium. In this way, all the individuals had the same available resources [23]. Before the treatment, all seedlings were watered daily to maintain robust growth.

### Watering treatments

The watering treatments started on July 7, five weeks after sowing. The watering heterogeneity treatment had four levels of water supply frequency: watering every 6 days (W1), watering every 3 days (W2), watering every 2 days (W3), and watering daily (W4). For each of these levels, the same amount of water was supplied for every cycle (6 days). In the mixed culture, the volume of added water was 600 ml for 2 cycles, 900 ml for the following 2 cycles, and 1,200 ml for another 6 cycles. The amount of water added in the monoculture in each watering event was half of that added in the mixed culture. No water loss due to drainage from the pots was observed during the experiment. There were 10 replicated pots for each treatment, and thus, there were 120 pots in total. All pots were watered at 18:00 (local time) and the watering treatment lasted 60 days. The pots were randomly arranged in the greenhouse, and rearranged weekly to minimize the potential influence of environmental gradients in the greenhouse.

### Measurements and calculations

#### Soil water content

**Fig 1:**
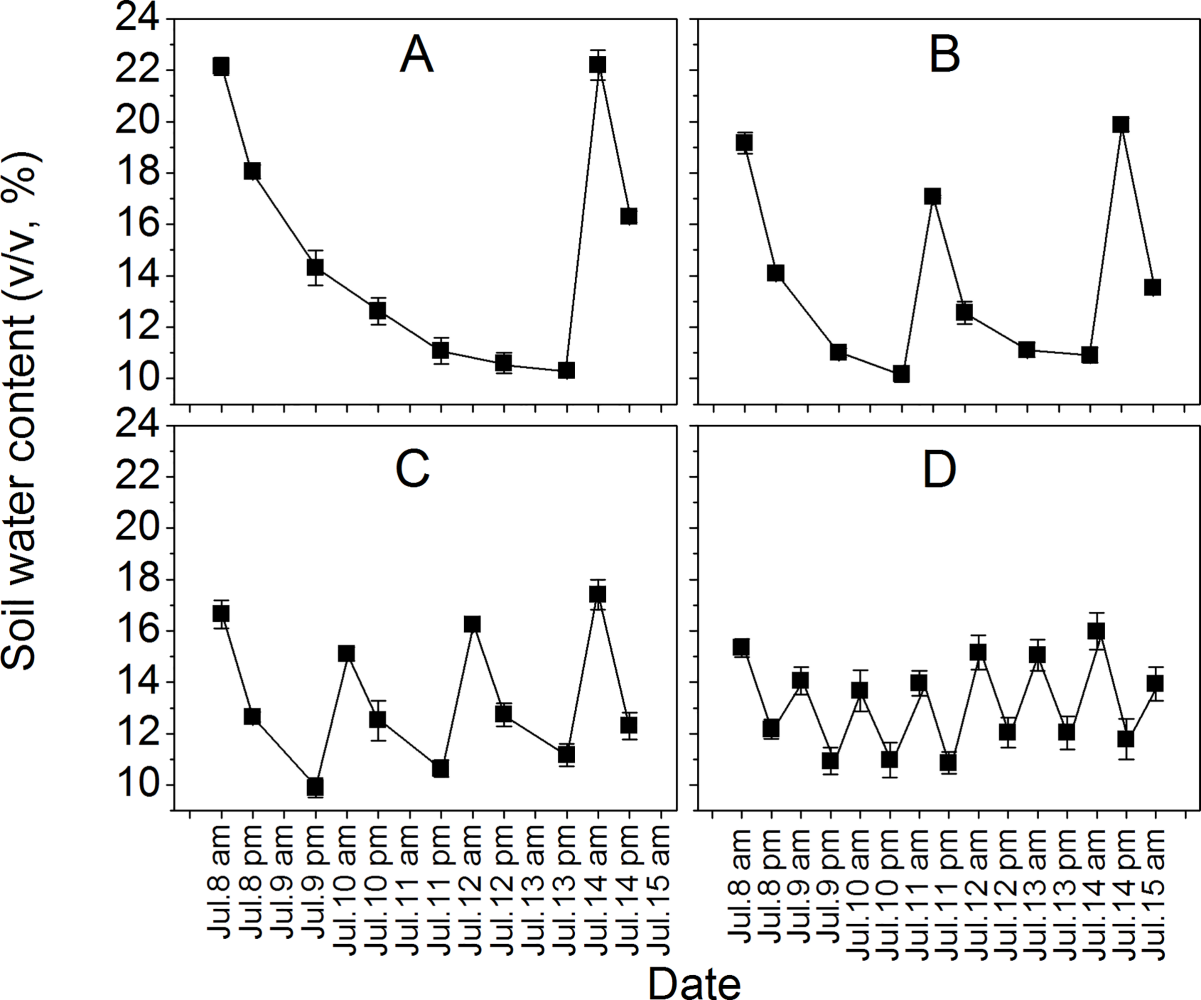
Soil water content (v/v, %) in different watering treatments. Water content in the mixed culture treatment was present. More than one cycle of watering treatments (from July 8 to 15) is shown. (A) W1 treatment; (B) W2 treatment; (C) W3 treatment; (D) W4 treatment. Values are means ± SE (n = 3), and were measured at 7:00–8:00 am and 17:00–18:00 pm.

During the experiment, soil water content (by volume) was monitored at a depth of about 10 cm, where plant roots were mainly distributed, with a soil moisture probe (WET Sensor, Delta-T Devices Ltd., Cambridge, UK). The watering treatment effects were shown with the mixed culture treatment as representative. In the monoculture, the soil water content trend was the same as in the mixed culture (data not shown). For the measurements, three pots were selected for each treatment and the results are demonstrated in Fig 1.

#### Leaf chlorophyll fluorescence

Chlorophyll fluorescence was measured using a pulse amplitude modulation chlorophyll fluorometer (Mini-PAM, Walz GmbH, Effeltrich, Germany). Five fully elongated leaves in the upper layer from five seedlings were selected for each treatment, and were dark-adapted with leaf clips for 30 min to ensure complete relaxation of all reaction centers before measurements on a sunny day. The minimum chlorophyll fluorescence (F_0_) was determined using a measuring beam, whereas the maximum chlorophyll fluorescence (F_m_) was recorded after 0.8 s of saturating pulse light (about 8,000 μmol m^-2^s^-1^). The maximal quantum yield of PSII (F_v_/F_m_) was calculated as (F_m_-F_0_)/F_m_ [25, 26].

#### Leaf chlorophyll content

At the end of the experiment, five healthy leaves for each treatment were selected for the chlorophyll content assay. Leaf discs of 1 cm^2^ were cut and both fresh weights and leaf areas were determined. Then, the leaf disks were soaked in 10 ml 95% (v/v) ethanol for about 48 h. Absorbance of the extracts was measured spectrophotometrically at wavelengths of 665 and 649 nm to measure the concentrations of chlorophyll a (C_a_) and chlorophyll b (C_b_) as described by Lichtenthaler and Wellburn (1983) [27]. Total chlorophyll content (Chl_total_) and chlorophyll a to chlorophyll b ratios (C_a_/C_b_) were also calculated. Chlorophyll concentrations were based on both the leaf weight and leaf area.

#### Leaf morphological traits

At the end of the watering treatments, 10 healthy mature leaves in each treatment were removed. The leaf images were obtained with a scanner and analyzed using WinFOLIA software (Regent Instruments Inc., Quebec, Canada) before their dry mass (LDM) was determined after drying in an oven at 70°C for 48 h. Leaf lengths (LL), leaf widths (LW), petiole lengths (PL), leaf perimeters (LP), and leaf areas (LA) were acquired to calculate specific leaf weight (LMA, LDM/LA), LL/LW, LL/PL, and γ (LP^2^/LA).

#### Plant fitness

We used the definition of fitness as the relative ability of an individual to survive, reproduce and propagate genes in a given environment [28]. Therefore, in this research, the number of flowering and surviving individuals were recorded for each treatment at the end of the experiment. The individuals were considered dead when the whole plant became withered and brown to black in color.

#### Plant growth

Final plant height (H), basal diameter (BD, at about 1 cm above the ground), crown area (CA), and branch number (BN) were recorded. The CA was calculated based on the ellipse area formula: CA = πab/4, in which a and b were the length of long axis and short axis. Stem specific density (ρ_stem_) was determined based on an approximately 5-cm portion of the middle section of the stem. The stem segments were excised and their volumes were measured using the water-displacement method, before oven drying at 70°C for 48 h to obtain dry mass. The ρ_stem_ was calculated as stem dry mass divided by volume.

After that, all seedlings of both species were harvested and divided into roots, stems, and leaves. Leaves were scanned and individual total leaf areas (TLA) were analyzed using WinFOLIA software. Roots were washed thoroughly and carefully with tap water. Roots of different species can be easily distinguished by their color in the mixed cultures. During the experiment, the fallen leaves were also collected. The plant parts were dried at 70°C for 48 h and weighed afterwards. Then the following parameters were calculated [29]:

Totalbiomass=rootbiomass+stembiomass+leafbiomass (1)

Rootmassratio(RMR)=rootbiomass/totalbiomass (2)

Stemmassratio(SMR)=stembiomass/totalbiomass (3)

Leafmassratio(LMR)=leafbiomass/totalbiomass (4)

Roottoshootmassratio(R/S)=rootbiomass/(stembiomass+leafbiomass) (5)

Fallenleafratio(FLR)=fallenleavesbiomass/totalleavesbiomass (6)

Plantheighttoabovegroundbiomassratio(HMR)=height/(stembiomass+leafbiomass) (7)

#### Relative dominance index

The relative dominance index (RDI) of *R*. *typhina* and *V*. *negundo* var. *heterophylla* was assessed in the mixed culture treatment with the following formula [8]:

RDI=biomassofonespecies/totalbiomassoftwospeciesinapot

Above- and below-ground competition was calculated separately, as there are fundamental differences between shoot and root competition (one is size-asymmetric and the other is symmetric) [30].

#### Traits plasticity index

The plasticity index (*PI*) was calculated as the difference between the maximum and minimum values divided by the maximum value over treatments modified from Godoy et al. (2011) [31]. *PI* represents the plasticity for a trait in different environments, which ranges from zero (minimum plasticity) to one (maximum plasticity). *PI* in mixed culture and monoculture treatments was calculated separately in both species.

### Statistical analysis

Data for each species were subjected to a separate two-way analysis of variance (ANOVA) where watering treatment and cultivation were considered as fixed factors. The variables were tested for normality and homogeneity of variance and were log- or sqrt-transformed when necessary. One-way ANOVA and the Tukey’s Honestly Significant Difference test were performed to determine the treatment differences in each species. A difference was considered to be statistically significant at *P* = 0.05 in all the tests. Plant fitness was compared using the Chi-square test, in which the likelihood ratio method was selected. All statistical analyses were performed using SPSS 13.0 software (SPSS Inc. Chicago, Illinois, USA).

## Results

### Soil water content

Soil water content varied between 10 and 23% during our experiment (Fig 1), and the variation was reduced from the W1 to W4 treatment. Although there was a decreasing trend of mean water content from the W1 to W4 treatment, no significant difference was found (*P* = 0.43).

### Leaf traits

**Fig 2:**
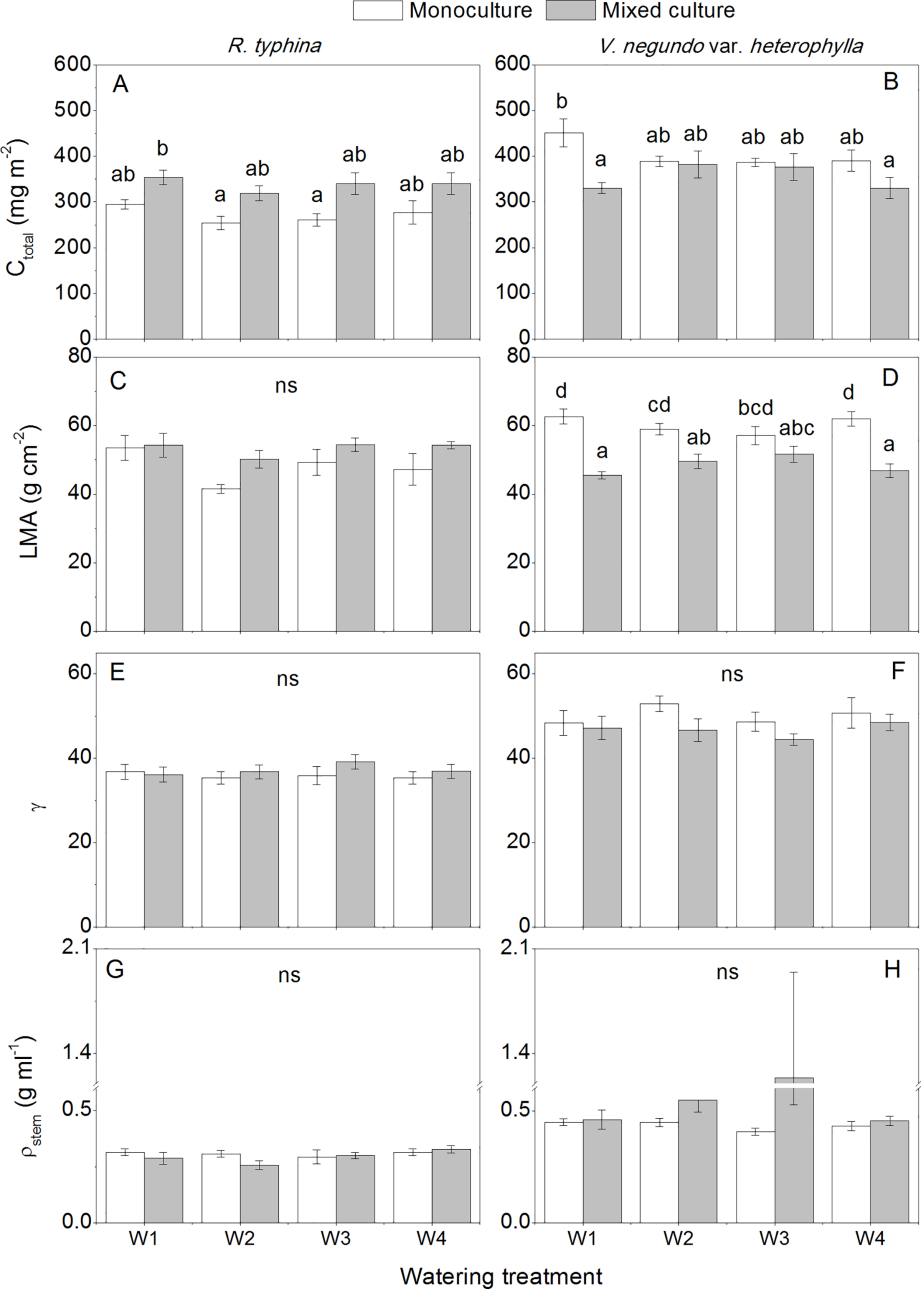
Leaf and stem traits of *Rhus typhina* and *Vitex negundo* var. *heterophylla* with different watering and cultivation treatments. C_total_ Total chlorophyll content, LMA Specific leaf weight, γ The ratio of square of leaf perimeter to leaf area, ρ_stem_ Stem specific density. The different lower case letters indicate significant differences between the treatments for each species and *ns* indicates no significant treatment effects (*P* ≤ 0.05, Tukey’s test). Bars are means ± SE (n = 5 for C_total_, n = 10 for LMA and γ, and n = 6–10 for ρ_stem_).

**Fig 3:**
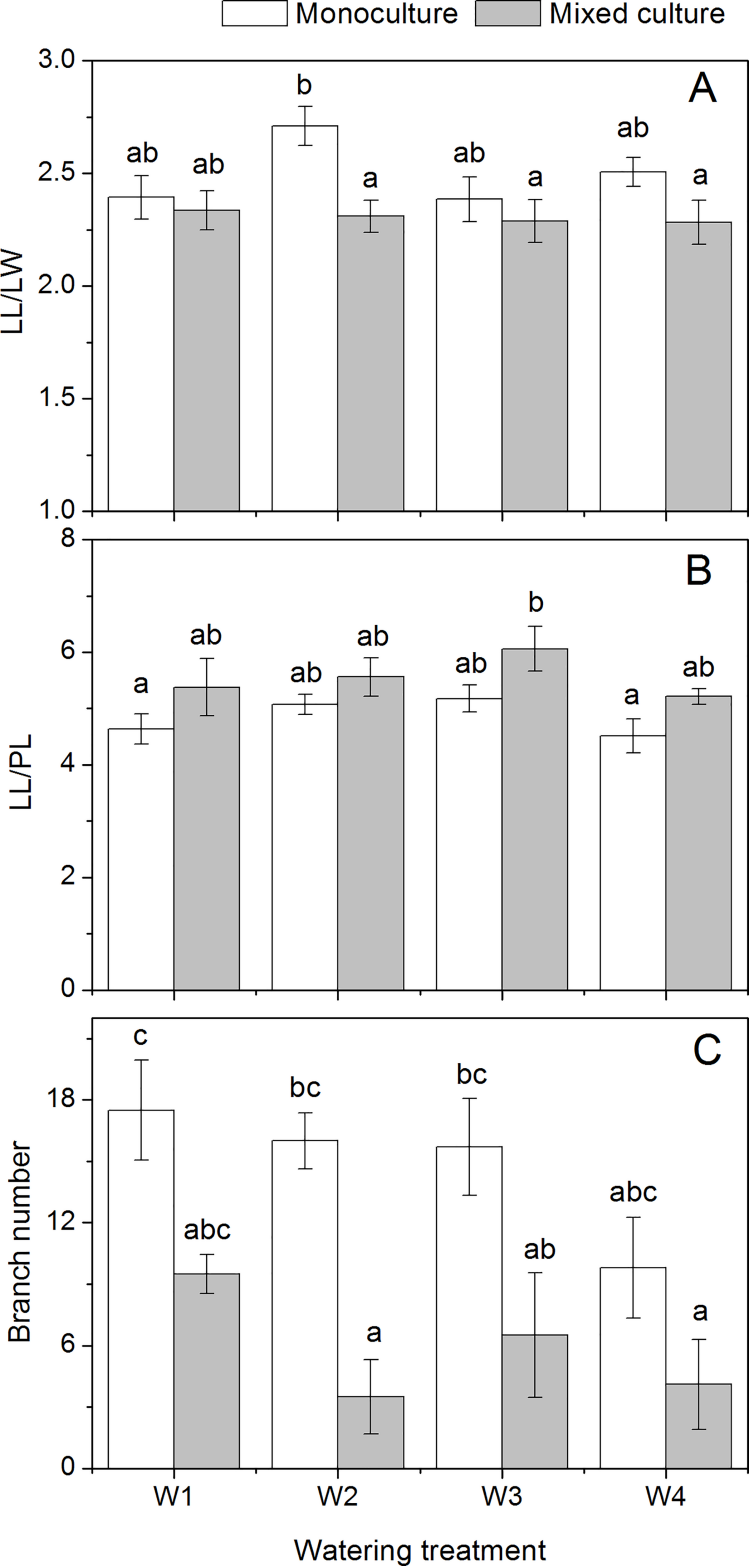
Leaf and stem morphological traits of *Vitex negundo* var. *heterophylla* with different watering and cultivation treatments. LL/LW The ratio of leaf length to leaf width, LL/PL The ratio of leaf length to petiole length. The different lower case letters indicate significant differences between the treatments (*P* ≤ 0.05, Tukey’s test). Bars are means ± SE (n = 10 for LL/LW and LL/PL, n = 6–10 for branch number).

Both leaf physiological and morphological traits were measured. Generally, the impact of cultivation treatment was more significant than the watering treatment (Table 1). In *R*. *typhina*, leaf chlorophyll content was higher in the mixed culture than in the monoculture treatment (Fig 2A), whereas a decreasing trend was present in *V*. *negundo* var. *heterophylla* based on the leaf area (Fig 2B). The other leaf traits of *R*. *typhina*, including C_a_/C_b_, F_v_/F_m_, LMA, LL/LW, and γ showed little variation among different treatments (Table 1). In comparison, the native species grown in mixed culture exhibited more morphological plasticity, including lower LMA, LL/LW, and γ values, and higher LL/PL values (Figs 2 and 3). In addition, LMA was also affected by the interaction of cultivation and watering treatment in *V*. *negundo* var. *heterophylla* (Table 1).

**Table 1:** The proportion of explained variance for functional traits illuminated separately for *Rhus typhina* and *Vitex negundo* var. *heterophylla* with cultivation (C) and watering treatment (W) as fixed main effects and their interactions.

Functional traits	*Rhus typhina* Source of variation (df)				*Vitex negundo* var. *heterophylla* Source of variation (df)			
	Cultivation (1)	Watering (3)	C×W (3)	*R*^2^	Cultivation (1)	Watering (3)	C×W (3)	*R*^2^
**Leaf traits**								
C_total_ (mg g^-1^)	15.0*	5.4^ns^	2.6^ns^	23.0	0.06^ns^	4.6 ^ns^	12.0^ns^	16.7
C_total_ (mg m^-2^)	40.4***	6.8 ^ns^	0.5 ^ns^	47.8	18.4**	3.9 ^ns^	16.1 ^ns^	38.5
C_a_/C_b_	2.4^ns^	7.4^ns^	15.0^ns^	27.2	2.0^ns^	4.9^ns^	15.0^ns^	21.9
F_v_/F_m_	1.8 ^ns^	6.1 ^ns^	0.1^ns^	7.9	1.9 ^ns^	5.8 ^ns^	7.1 ^ns^	14.8
LMA (g m^-2^)	7.3*	8.7^ns^	2.2^ns^	18.2	44.2***	0.02^ns^	6.9*	51.1
LL/LW	2.9^ns^	2.5^ns^	3.5^ns^	8.9	10.8 **	4.9^ns^	5.1 ^ns^	20.8
LL/PL	—	—	—	—	11.0**	7.5 ^ns^	0.4 ^ns^	19.0
γ	1.8^ns^	1.2^ns^	1.7^ns^	4.7	4.8 ^ns^	2.9 ^ns^	1.5 ^ns^	9.1
**Stem traits**								
Branch number	—	—	—	—	26.6***	7.6^ns^	2.1 ^ns^	36.3
ρ_stem_ (g ml^-1^)	1.3 ^ns^	4.4^ns^	4.0 ^ns^	9.7	2.0^ns^	3.7^ns^	4.4 ^ns^	10.1
**Plant Growth and Partitioning**								
BD (mm)	36.4***	1.2^ns^	1.6^ns^	39.3	61.1***	0.6 ^ns^	0.3 ^ns^	62.0
H (cm)	14.4***	0.9^ns^	0.5^ns^	15.8	48.5***	0.4^ns^	2.7 ^ns^	51.6
CA (cm^2^)	2.3ns	3.4^ns^	5.1^ns^	10.8	46.8***	0.7 ^ns^	5.5 ^ns^	53.1
TLA (cm^2^)	13.5***	2.7^ns^	2.2^ns^	18.5	74.7***	0.2^ns^	1.3 ^ns^	76.2
Root biomass (g)	50.8***	5.2*	6.6**	62.6	74.3***	0.2^ns^	0.2 ^ns^	74.7
Stem biomass (g)	36.1***	1.0 ^ns^	0.3 ^ns^	37.4	57.0***	0.1 ^ns^	2.9 ^ns^	60.0
Leaf biomass (g)	31.9***	1.1 ^ns^	2.6 ^ns^	35.6	79.2***	0.02 ^ns^	1.3 ^ns^	80.5
Total biomass (g)	54.2***	2.2 ^ns^	1.9 ^ns^	58.3	80.5***	0.09^ns^	0.9 ^ns^	81.5
RMR	4.2 ^ns^	6.5 ^ns^	8.3 ^ns^	19.0	10.9**	7.3 ^ns^	14.4**	32.5
SMR	3.6 ^ns^	1.2 ^ns^	4.6 ^ns^	9.4	0.4^ns^	2.6 ^ns^	11.0 ^ns^	27.6
LMR	11.8**	6.5 ^ns^	9.7 *	28.0	18.7***	7.5 ^ns^	10.5*	36.7
R/S	4.2 ^ns^	6.9 ^ns^	6.3 ^ns^	17.3	12.7***	8.1^ns^	13.1 **	33.9
FLR	36.4***	0.4 ^ns^	0.7 ^ns^	37.4	12.4**	2.0 ^ns^	1.8 ^ns^	16.3
HMR (m g^-1^)	24.4***	6.0 ^ns^	11.7**	42.1	47.8***	2.4 ^ns^	2.5 ^ns^	52.7

The proportion of explained variance (SS_x_/SS_total_) for each factor and the interactions are indicated. ^ns^: *P* > 0.05

*: P ≤ 0.05

**: P ≤ 0.01

***: P ≤ 0.001.

Degrees of freedom (df) are also indicated. *R*^2^ is the proportion of total variance absorbed by the model. Monoculture and mixed culture treatment are included in the cultivation effects, and four levels of frequency of water supply are included in the watering treatment.

C_total_ Total chlorophyll content, C_a_/C_b_ Ratio of chlorophyll a to b, F_v_/F_m_ Maximal quantum yield of PSII, LMA Specific leaf weight, LL/LW The ratio of leaf length to leaf width, LL/PL The ratio of leaf length to petiole length, γ The ratio of square of leaf perimeter to leaf area, ρ_stem_ Stem specific density, BD Basal diameter, H Plant height, CA Crown area, TLA Individual total leaf areas, RMR Root mass ratio, SMR Stem mass ratio, LMR Leaf mass ratio, FLR Fallen leaf ratio, R/S The ratio of root biomass to shoot biomass, HMR The ratio of plant height to aboveground biomass.

LL/PL and branch number were not measured in *R*. *typhina* as the petiole was too short to measure and the plants did not produce branches.

### Stem traits

In our experiment, branch numbers (BN) and stem specific density (ρ_stem_) were recorded to indicate the species’ acclimation to cultivation and watering treatment regarding shoot growth strategies. BN of *V*. *negundo* var. *heterophylla* was significantly reduced in the mixed culture treatment in all watering treatments (Fig 3C). The ρ_stem_ in both species showed little variation, i.e., cultivation, watering treatment or their interaction had no significant effect (Fig 2G and 2H). By comparison, ρ_stem_ was lower in *R*. *typhina* than in *V*. *negundo* var. *heterophylla* in all watering and cultivation treatments.

### Plant fitness

The effect of cultivation and watering treatment on plant fitness was shown in Table 2. No *R*. *typhina* plants were found to bloom in any of the cultivation and watering treatments. In the case of *V*. *negundo* var. *heterophylla*, there was an increasing trend for flowering from W1 to W4 in the monoculture treatment, whereas the trend was not significant in the mixed culture treatment. The difference between monoculture and mixed culture was only significant in the W4 treatment. The number of dead plants of *V*. *negundo* var. *heterophylla* was both cultivation and watering treatment-dependent (Table 2). The largest mortality rate existed in the mixed culture and W1 treatment, in which the water was supplied with low frequency.

**Table 2:** Plant fitness of *Rhus typhina* and *Vitex negundo* var. *heterophylla* under different cultivation and watering treatments.

Index	Watering treatment	*R*. *typhina*		*V*. *negundo* var. *heterophylla*	
		Monoculture	Mixed culture	Monoculture	Mixed culture
No. of flowering individuals	W1	0	0	1	2
	W2	0	0	2	1
	W3	0	0	2	3
	W4	0	0	**3**	**0**
No. of dead individuals	W1	0	0	**0**	**4**
	W2	0	1	1	2
	W3	0	0	0	0
	W4	0	0	0	0

W1 watering every 6 days, W2 watering every 3 days, W3 watering every 2 days, and W4 watering daily. The same total amount of water was supplied in different watering heterogeneity treatments. Significance between monoculture and mixed culture is indicated by bold font and underlines (Chi-square test, *P* < 0.05).

### Plant growth and partitioning

**Fig 4:**
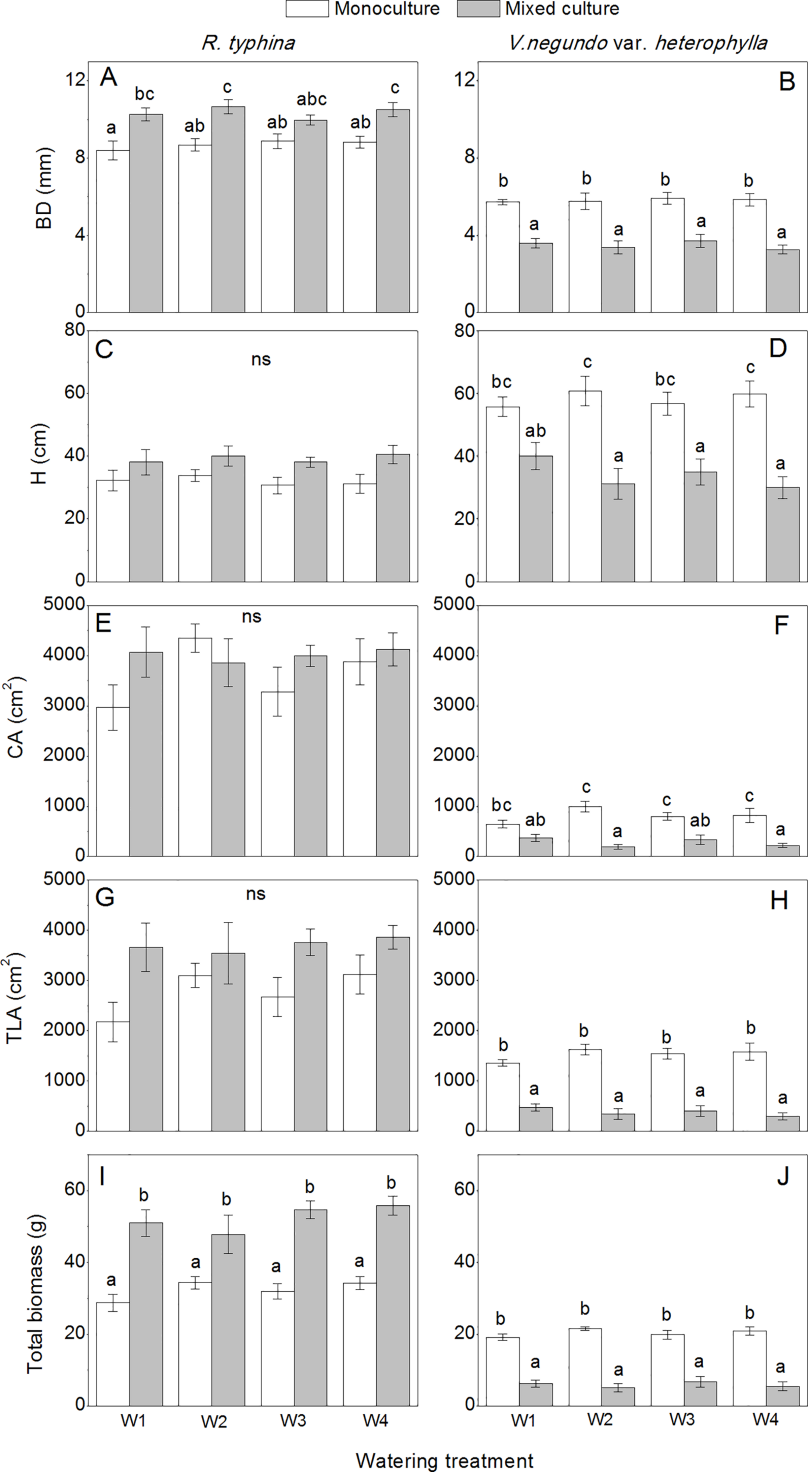
Plant growth of *Rhus typhina* and *Vitex negundo* var. *heterophylla* with different watering and cultivation treatments. BD Basal diameter, H Plant height, CA Crown area, TLA Individual total leaf areas. The different lower case letters indicate significant differences between the treatments for each species and *ns* indicates no significant treatment effects (*P* ≤ 0.05, Tukey’s test). Bars are means ± SE (n = 6–10).

There was no effect of water treatment on plant growth in both species (Table 1), except for root biomass in *R*. *typhina*. Irrespective of watering treatment, the growth of *R*. *typhina* increased, whereas that of *V*. *negundo* var. *heterophylla* decreased in the mixed culture treatment than that of the monoculture treatment (Fig 4). On average, the BD and H in the mixed culture increased by about 20% in *R*. *typhina*, but decreased by about 70% in *V*. *negundo* var. *heterophylla*. Mixed culture cultivation treatment resulted in a 13.5 and 36.8% increase of CA and TLA in *R*. *typhina*, and 225.8 and 329.2% reduction in *V*. *negundo* var. *heterophylla*, respectively. The plant biomass of *R*. *typhina* in mixed culture was not more than twice that in monoculture, whereas the biomass of *V*. *negundo* var. *heterophylla* in monoculture was three to four times higher than that of the mixed culture cultivation treatment. Overall, the growth parameters of *V*. *negundo* var. *heterophylla* were more affected than the other species by the mixed culture treatment.

**Fig 5:**
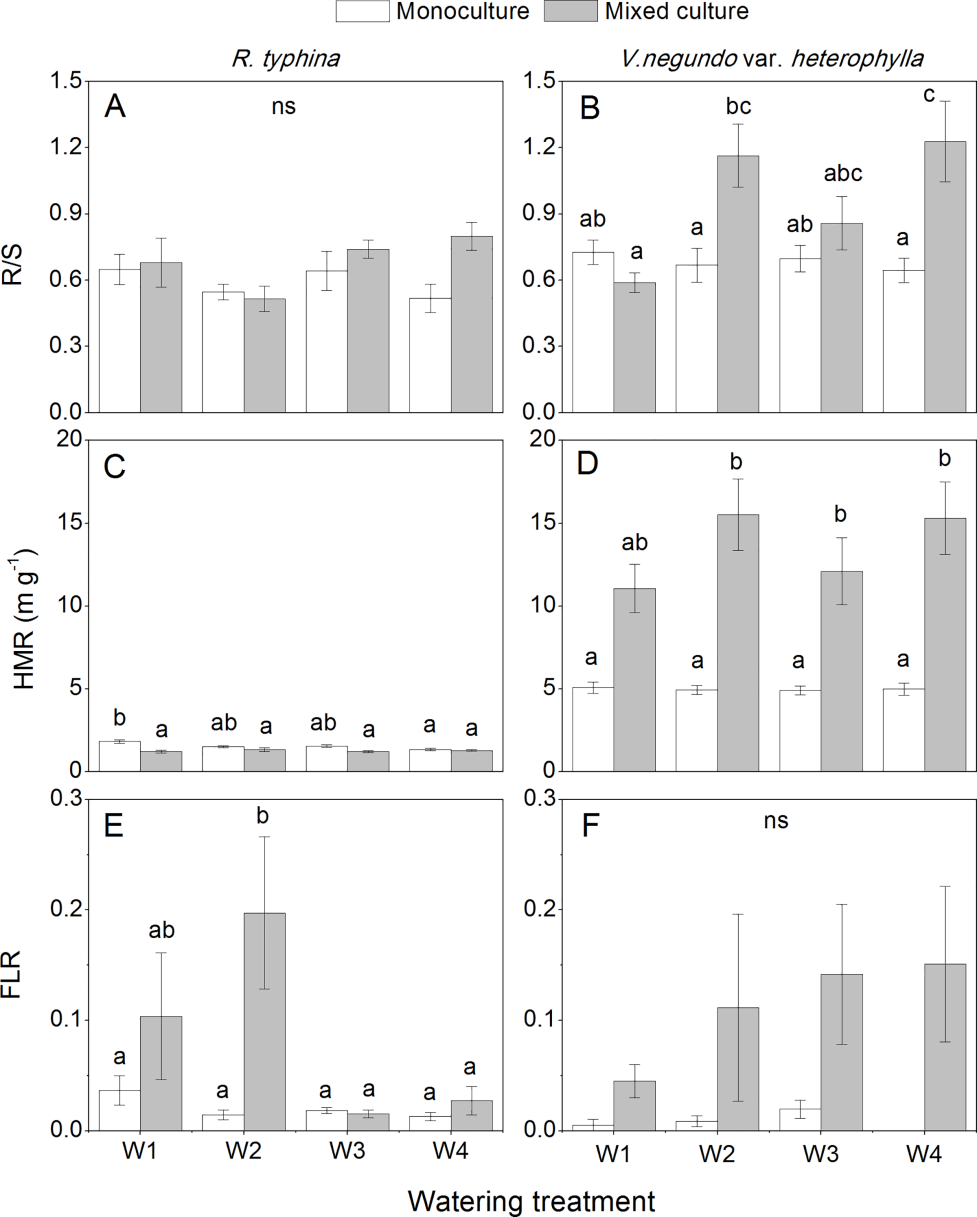
Plant biomass partitioning of *Rhus typhina* and *Vitex negundo* var. *heterophylla* with different watering and cultivation treatments. R/S The ratio of root to shoot biomass, HMR The ratio of plant height to aboveground biomass, FLR Fallen leaf ratio. The different lower case letters indicate significant differences between the treatments for each species and *ns* indicates no significant treatment effects (*P* ≤ 0.05, Tukey’s test). Bars are means ± SE (n = 6–10).

SMR was stable in our experiment in both species, which indicated that the adjustment of biomass allocation was mainly between leaves and roots. Compared with the monoculture treatment, plants in the mixed culture treatment showed higher RMR and lower LMR, which resulted in a higher R/S (Fig 5B). Specifically, the native species exhibited higher R/S than the alien species, and this distinction was more significant in the mixed culture treatment (Fig 5A and 5B).

HMR was also cultivation-dependent in both species, and the variation was more notable in the native species (Fig 5C and 5D). By contrast, HMR in *R*. *typhina* was much lower than that in *V*. *negundo* var. *heterophylla* regardless of the cultivation and watering treatment.

ANOVA revealed significant effects of cultivation treatment on the FLR in both species (Table 1). In the mixed culture treatment, the increase of FLR was noticeable in the W1 and W2 treatments in *R*. *typhina*. Nevertheless, FLR tended to be higher in the mixed culture treatment in *V*. *negundo* var. *heterophylla* in all watering treatments (Fig 5E and 5F).

### Comprehensive analyses of the plant characteristics

**Fig 6:**
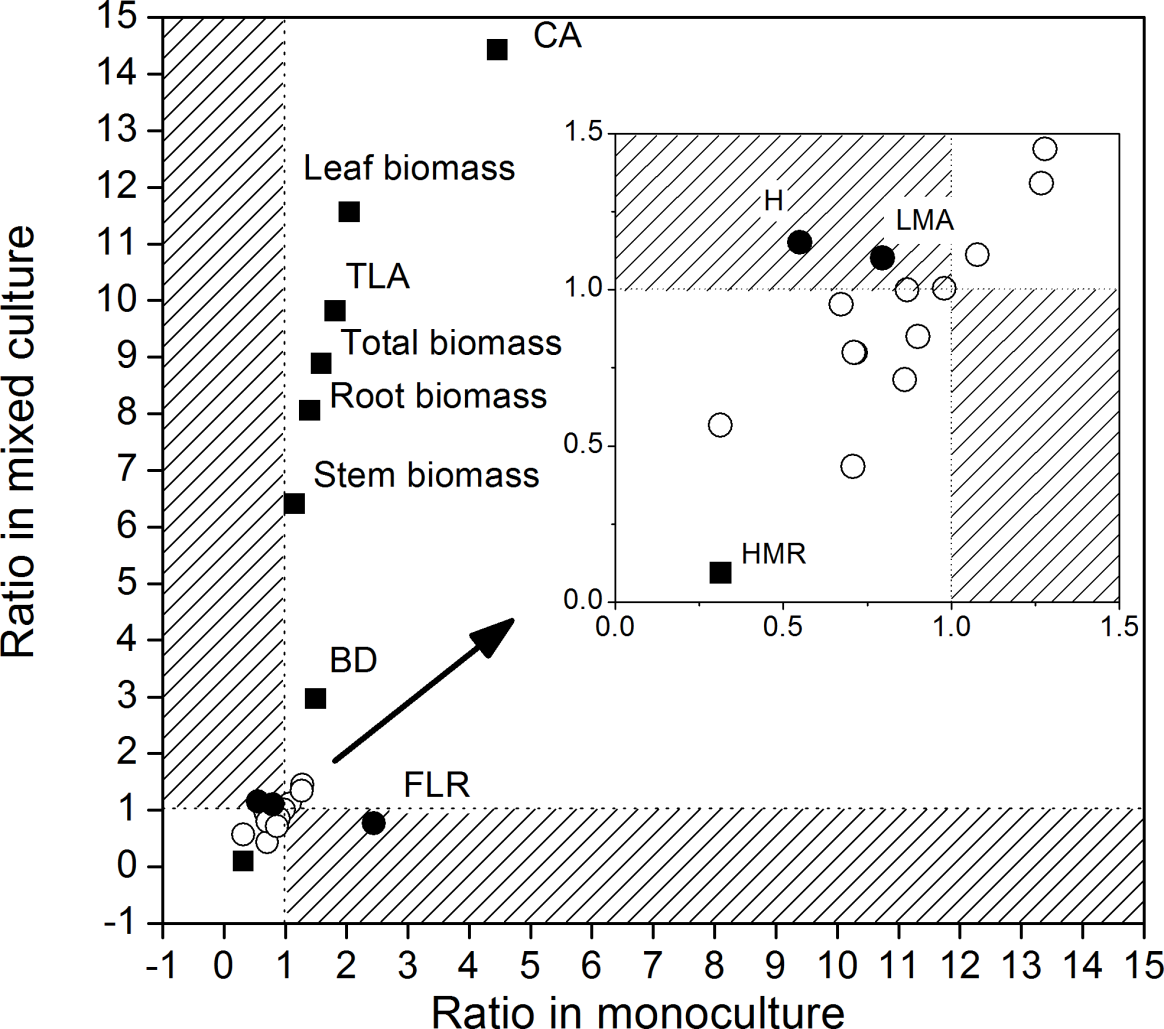
A schematic representation of the functional traits ratio between *Rhus typhina* and *Vitex negundo* var. *heterophylla* in monoculture and mixed culture treatments. The crowded points were enlarged on the top right corner. LMA Specific leaf weight, BD Basal diameter, H Plant height, CA Crown area, TLA Individual total leaf areas, FLR Fallen leaf ratio, HMR The ratio of plant height to aboveground biomass. The areas with diagonal stripes refer to the “reversal region”, where the trait values have an opposite trend in monoculture and mixed culture treatments. Traits located in the “reversal region” are indicated by filled circle points; traits that have the same trends but show two-fold or greater differences in monoculture and mixed culture treatments are indicated by filled square points.

Plant traits were further analyzed using the ratio calculation method, which was used to assess relative values between the two species with competition. As the watering treatment effect was not significant, plant functional traits in all water regimes were averaged in monoculture and mixed culture treatments separately for both species. Then, the ratios of *R*. *typhina* to *V*. *negundo* var. *heterophylla* in monoculture and mixed culture were calculated. H, LMA, and FLR were located in the “reversal region” (areas with the diagonal stripes in Fig 6), which indicates that the trait values of *R*. *typhina* and *V*. *negundo* var. *heterophylla* had the opposite trend in monoculture and mixed culture treatments. Although CA, leaf biomass, TLA, total biomass, root biomass, stem biomass, BD, and HMR of the two species had the same trends in monoculture and mixed culture, the difference was greater than two times.

### Trait plasticity index

**Fig 7:**
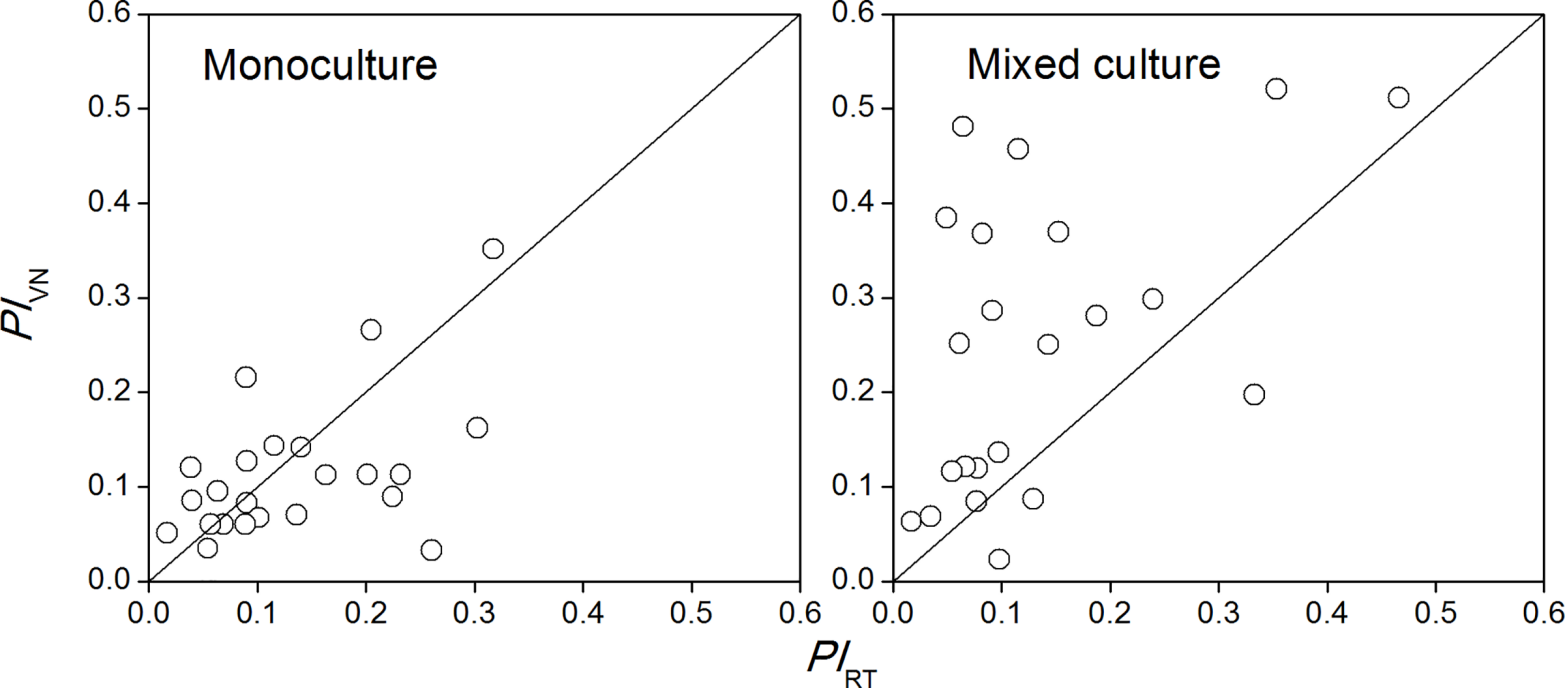
Trait plasticity index of *Rhus typhina* (*PI*_RT_) and *Vitex negundo* var. *heterophylla* (*PI*_VN_) in monoculture and mixed culture.

The trait plasticity index (*PI*) of different water conditions is illustrated in Fig 7. Twenty-two plant traits are included in the figure. For conciseness, the names of plant traits are not shown in the *PI* figure. In the monoculture treatment, the number of *PI*_VN_ > *PI*_RT_ and *PI*_RT_ > *PI*_VN_ was similar. However, in the mixed culture treatment, most of the *PI*s were higher in the native species.

### Relative dominance index

The RDI of the two species grown in mixed culture treatments were calculated in Table 3. RDIs of *RT* were significantly larger than that of *VN*, indicating that *R*. *typhina* showed absolute dominance to *V*. *negundo* var. *heterophylla* both above and below ground. In addition, the RDI did not show significant differences in different watering treatments.

**Table 3:** Relative dominance index (RDI) of *Rhus typhina* (*RT*) and *Vitex negundo* var. *heterophylla* (*VN*) in mixed cultures for four different watering treatments.

Watering treatment	Above-ground		Below-ground	
	RDI of *RT*	RDI of *VN*	RDI of *RT*	RDI of *VN*
W1	0.898±0.017	0.102±0.017	0.903±0.014	0.097±0.014
W2	0.922±0.024	0.078±0.024	0.847±0.036	0.153±0.036
W3	0.890±0.027	0.110±0.027	0.887±0.022	0.113±0.022
W4	0.917±0.024	0.083±0.024	0.898±0.023	0.102±0.023

Values are mean ± SE (n = 6–10), the pots with dead plants are not included in the table.

## Discussion

### Competition and acclimation of the two species

Our results showed that *R*. *typhina* was the superior competitor in the mixed culture treatment, which was especially obvious regarding crown area, stem basal diameter, total leaf area, and plant biomass (Figs 4 and 6). In addition, the competition ability was not increased under heterogeneous watering treatments (Table 3), which did not support our hypothesis that *R*. *typhina* would be more superior under fluctuating environmental conditions. The dominance of *R*. *typhina* appeared to be mainly derived from its considerable growth ability and final above and below ground biomass. *R*. *typhina* had a growth strategy with a large crown area and stem basal diameter (Fig 4), and the leaf lateral expansion (higher crown area and total leaf area) were always fast, which is beneficial to its competition for sunlight [19]. Plant height growth was not the main factor to determine competition success in our study, although height is usually the key factor in determining the success of light competition between individuals in other species [9]. *R*. *typhina* could also maintain much higher root biomass under competition, which was also supported by Yuan et al. (2013) [8], and the higher root biomass resulted from a high carbon assimilation capacity of *R*. *typhina* [8]. Higher root biomass is beneficial to plants to compete for water and nutrients. The higher below-ground competition ability was also confirmed by another alien species, *Robinia pseudoacacia*, which hampered the growth of *Q*. *acutissima* by decreasing soil resources availability [9]. However, an effective pre-emption of above and below ground resources is critical for alien species performance under competition to suppress the growth of neighbors.

Plant growth superiority is strongly related to several leaf, stem, and root traits. It may be possible to predict future plant invasions from species traits [5, 6, 32]. In our study, chlorophyll content of the alien species was higher in the mixed culture treatment, which was advantageous for light capture under competition. Besides, the rapid growth ability of *R*. *typhina* can be partly derived from its low stem density compared that of to the native species, since plants with high growth rate may show low stem density [33]. Stem density is also regarded as critical in a trade-off between plant growth (low stem density) and stem defenses by biotic and abiotic factors (high stem density) [33]. Rapid initial growth would be crucial to the preponderant status of *R*. *typhina*, since larger seedlings in forest gaps are more likely to survive and eventually reach the canopy [29].

High phenotypic plasticity enhances the species’ ecological niche breadth and allows organisms to grow efficiently in a broader range of environments [34], so alien species, especially invasive species, may demonstrate significantly higher phenotypic plasticity than native ones [28, 34]; but, this was not the case in our study. The trait plasticity index was similar between the two species, and a higher plasticity index was found in *V*. *negundo* var. *heterophylla* than *R*. *typhina* in the mixed culture treatment (Fig 7). This indicated that the competition superiority of *R*. *typhina* did not depend on a higher plasticity index for water regime, and there was a strong acclimation capacity of the native species to cultivation and water supply patterns. However, a given trait may be plastic in a certain set of environments, but not plastic in a different set of environments [34]. Our results were only obtained under different water treatments; therefore, the phenotypic plasticity of *V*. *negundo* var. *heterophylla* and *R*. *typhina* needs to be further investigated in nature to fully evaluate the invasiveness of the alien shrub species.

*V*. *negundo* var. *heterophylla* exhibited acclimations to competition from different aspects. Apart from some individuals of *V*. *negundo* var. *heterophylla* that died owing to competition, the surviving plants showed notable adjustment ability and conservation mechanisms. In the mixed culture, a lower specific leaf weight (LMA), leaf length to leaf width ratio (LL/LW), and higher leaf length to petiole length ratio (LL/PL) were found in *V*. *negundo* var. *heterophylla*. Obviously, these regulations were beneficial for the species to absorb irradiance effectively within the shade of alien species. The results were consistent with our previous research [17], and it was an effective spatial plant component arrangement to absorb light for the species. *V*. *negundo* var. *heterophylla* individuals showed a higher rate of flowering in monoculture (Table 2), which suggests that it had higher fitness. As a native species, *V*. *negundo* var. *heterophylla* must have adapted the local environment since selection always maximizes fitness in a given environment [28]. The plant height of *V*. *negundo* var. *heterophylla* was comparable to that of *R*. *typhina* in the mixed culture, which is due to the acclimation for enhancing light capture. The strategy was further confirmed by the plant height to aboveground biomass ratio (HMR), which became substantially higher under low irradiance [29]. In competition with *R*. *typhina*, although the absolute root biomass was much lower, *V*. *negundo* var. *heterophylla* allocated relatively more biomass to roots (higher root biomass to shoot biomass ratio [R/S]), which is a strategy to compete for underground resources. A higher R/S was also detected in another native species *Q*. *acutissima*, when it was grown with an alien species *R*. *pseudoacacia* [9]. In brief, the ability of plants to acclimate their functional traits to different environments, including the high phenotypic plasticity in competition, are important reasons for the wide distribution of *V*. *negundo* var. *heterophylla* in northern China.

There can be expected trade-offs in species’ competition ability in pair-wise experiments. A superior competitor for one resource cannot be a superior competitor for all other resources [35]. In a species competition study with three grasses [16], the best competitors changed with water supply frequency. However, this was not the case in this experiment, since *R*. *typhina* showed absolute superiority in all the resource conditions. We also considered species identity as an important factor influencing competition outcomes. Our results were consistent with previously reported results [8], in which *R*. *typhina* also showed an absolute superiority over *Q*. *acutissima* in all N:P supply treatments.

### Effects of water supply treatment

Although water effect was not too large in our experiment, we cannot neglect its vital implication. Survival of *V*. *negundo* var. *heterophylla* in the mixed culture was dependent on water supply, and more individuals died in the W1 and W2 treatments (Table 2), which was quite different from the growth responses. Meanwhile, the fallen leaf ratio (FLR) of *R*. *typhina* was higher in a more heterogeneous water supply (Fig 5E), which indicates that the interaction of the two species may be more intense in this condition. Our results are consistent with the opinion of Sher et al. (2004) [13], who stated that mortality occurs during long periods of drought between resource renewal events, and this can be explained by the “two-phase resource hypothesis.” Obviously, the drought periods (interpulse length) in W1 and W2 treatments were longer than that in W3 and W4 treatments (Fig 1) in this research. More attention should be paid to this phenomenon since it may lead to species turnover in the field, and a similar pattern has been found in Kansas grasslands [36]. Nevertheless, temporal variability in soil moisture availability is as important as water supply quantity. As important components of global climate change, both aspects will determine the vegetation composition and dynamics in the future.

Generally, plants grow larger under a more homogeneous than heterogeneous water supply, regardless of the presence of neighbors, because plants can take up water more uniformly under homogeneous conditions [12, 16]. However, the results of species’ responses to water heterogeneity were inconsistent. In the study of Robinson and Gross (2010) [11], the biomass of some annual weed species was reduced with longer intervals, but some species showed either a positive or no response. In addition, most previous studies focused on grass species to study the effects of water temporal heterogeneity [10, 11, 12, 13], and studies with woody plant species are scarce. This is possibly another important reason for different results in our study. As the responses to water pulsing are species-specific and the different intrinsic attributes between grass and woody plant species, a longer treatment period is needed for woody plants. In the field, the focal species responses are also mediated by the surrounding community of competitors. The neighbors can either counter or augment species’ direct responses to altered resource supply. Which of these outcomes emerges depends on species differences in their direct physiological or demographic responses to environmental changes [37].

### Species dynamics and management in the future

Species invasion and management are important issues in the study of plant ecology. Alien species should be evaluated more comprehensively, using both scientific and social factors [19]. Our results highlighted the strong competitive ability of *R*. *typhina* with the native species, and the superiority will persist in different water supply patterns. This provided evidence that *R*. *typhina* may have a large impact on the species composition of local communities in the future. Together with its high light response plasticity [18] and absolute dominance over another dominant woody species, *Q*. *acutissima*, under different soil nutrient conditions [8], the authors urge more caution when introducing *R*. *typhina* in the context of global climate change. Our study further suggests that caution should be exercised for the use of *R*. *typhina* in revegetation activities [18].

However, the native species *V*. *negundo* var. *heterophylla* demonstrated good acclimation capacity to the competition and resource supply with various plant traits. In the field, significantly higher species richness, individual density and diversity were observed in the native *V*. *negundo* var. *heterophylla* community than in the *R*. *typhina* community at both sterile and fertile habitats [3]. In comparison, the native species *V*. *negundo* var. *heterophylla* will be more appropriate for revegetation. Similarly to the proposal of Wang et al. (2013) [19], ceasing the utilization of *R*. *typhina* in afforestation and finding an alternative native species may be a better choice.

The success of species invasion varies across habitats and timescales [2]. The naturalization period of *R*. *typhina* in Europe is longer than one hundred years, and the species was found to be difficult to control once it became invasive. Root sprouting is apparently stimulated by top-damage, and seedlings or small plants should only be hand-pulled [21]. In China, the period of introduction is no more than 60 years. It is possible that large impacts have not appeared yet, as the lag phases may exceed 100 years between the introduction and commencement of invasion [19, 38]. Based on more scientific research in China, a comprehensive risk evaluation system concerning both scientific and social factors for *R*. *typhina* should be developed and implemented in the future.

## Conclusions

In our study, the alien species *R*. *typhina* was the superior competitor in the mixed culture. In addition, the superiority did not change with water supply pattern. The dominance of *R*. *typhina* was mainly derived from its greater biomass and effective adjustment of leaf physiology. Higher chlorophyll content in mixed culture, and lower stem specific density relative to the native species conferred *R*. *typhina* with more rapid growth ability. *V*. *negundo* var. *heterophylla* exhibited higher leaf length to petiole length and root to shoot biomass ratios, and lower specific leaf weight and leaf length to leaf width ratio in competition. More individuals of *V*. *negundo* var. *heterophylla* died in low-frequency water supply treatments in competition with *R*. *typhina*. This phenomenon should receive more attention since it may lead to species turnover in the field. We urge more caution to introduce *R*. *typhina* in the context of global climate change.

## Supporting information

S1 Table Abbreviations of plant traits used in the study. (DOCX)

S1 Dataset Plant traits recorded in the study. (XLS)
