# Supplementary material for: Dominance of an alien shrub Rhus typhina over a native shrub Vitex negundo var. heterophylla under variable water supply patterns
Source: PLoS One. 2017 Apr 26;12(4):e0176491. doi: 10.1371/journal.pone.0176491 (PMC5406003; doi:10.1371/journal.pone.0176491)
Supplement: S1 Table — (DOCX) [file pone.0176491.s001.docx]

**S1 Table. Abbreviations of plant traits used in the study.**

| **Abbreviation** | **Full name** | **Calculation** |
| --- | --- | --- |
| F_0_ | The minimum chlorophyll fluorescence |  |
| F_m_ | The maximum chlorophyll fluorescence |  |
| F_v_/F_m_ | Maximal quantum yield of PSII | (F_m_-F_0_)/F_m_ |
| C_a_ | Concentrations of chlorophyll a |  |
| C_b_ | Concentrations of chlorophyll b |  |
| Chl_total_ | Total chlorophyll content | C_a_+ C_b_ |
| C_a_/C_b_ | Chlorophyll a to chlorophyll b ratio | C_a_/C_b_ |
| LDM | Leaf dry mass |  |
| LL | Leaf length |  |
| LW | Leaf width |  |
| PL | Petiole length |  |
| LP | Leaf perimeter |  |
| LA | Leaf area |  |
| LMA | Specific leaf weight | LDM/LA |
| LL/LW | The ratio of leaf length to leaf width | LL/LW |
| LL/PL | The ratio of leaf length to petiole length | LL/PL |
| γ | The ratio of square of leaf perimeter to leaf area | LP^2^/LA |
| H | Plant height |  |
| BD | Basal diameter |  |
| CA | Crown area |  |
| BN | Branch number |  |
| ρ_stem_ | Stem specific density | Stem dry mass/stem volume |
| TLA | Individual total leaf areas |  |
| RMR | Root mass ratio | Root biomass/total biomass |
| SMR | Stem mass ratio | Stem biomass/total biomass |
| LMR | Leaf mass ratio | Leaf biomass/total biomass |
| R/S | Root to shoot mass ratio | Root biomass/(stem biomass + leaf biomass) |
| FLR | Fallen leaf ratio | Fallen leaves biomass/total leaves biomass |
| HMR | Plant height to aboveground biomass ratio | Height/(stem biomass + leaf biomass) |
| RDI | Relative dominance index | Biomass of one species/total biomass of two species in a pot |
| PI | Plasticity index | (The maximum value-the minimum value)/the maximum value |
